# Life-threatening airway obstruction caused by angioedema in a morbidly obese postoperative patient: a case report

**DOI:** 10.1186/s40981-020-00408-6

**Published:** 2021-01-04

**Authors:** Makiko Konda, Satoki Inoue, Yusuke Naito, Junji Egawa, Masahiko Kawaguchi

**Affiliations:** Division of Intensive Care and Department of Anesthesiology, Nara Medical University, 840 Shijo-cho, Kashihara, Nara, 634-8522 Japan

**Keywords:** Angioedema, Angiotensin-converting enzyme inhibitor, Enalapril, Difficult airway

## Abstract

**Background:**

We report a case of a morbidly obese patient who developed life-threatening airway obstruction due to angioedema.

**Case presentation:**

A 50-year-old Japanese morbidly obese female was treated with enalapril for 10 years, with no history of angioedema. After 3 h of completion of breast cancer resection under general anesthesia with tracheal intubation, she developed airway obstruction and respiratory arrest. Her oral cavity was occupied with a swollen tongue. It was extremely difficult to determine the airway anatomical orientation although tracheal intubation was attempted using a videolaryngoscope. At this time, she probably started gasping respiration, which generated a faint bubble and revealed a possible airway. Her airway was established using a tracheal tube without confirming the glottis or the vocal cord.

**Conclusions:**

Angioedema induced by angiotensin-converting enzyme (ACE) inhibitors is rare; however, once it occurs, it can be potentially life threatening, especially for patients with possible difficult airway. Considering the risk–benefit ratio, we must be careful in administering ACE inhibitor therapy in morbidly obese patients.

## Background

Obesity tends to impair airway patency [1]. Therefore, it is obvious that airway edema can have a severer effect on airway patency in morbidly obese individuals than in lean individuals. Although angiotensin-converting enzyme (ACE) inhibitors have been extensively used for controlling hypertension, angioedema is one of the critical side effects. Herein, we report a case of a morbidly obese patient who developed life-threatening airway obstruction due to angioedema in the postoperative period, which was probably mediated by an ACE inhibitor.

## Case presentation

Written informed consent was obtained from the patient for publication of this case report and accompanying images. A 50-year-old Japanese female with a medical history of diabetes mellitus, chronic kidney disease (stage 4), and hypertension was admitted to our hospital for surgical removal of cancer in her left breast. She was morbidly obese (height = 160 cm, weight = 144 kg), had no history of asthma and allergy to any drugs or food, and had no memory of suffering from urticarial rashes. She had been taking daily doses of enalapril 10 mg, olmesartan 20 mg, amlodipine 5 mg, metoprolol 60 mg, trichlormethiazide 2 mg, and furosemide 20 mg for the management of her hypertension for approximately 10 years. On the day of surgery, she was treated only with amlodipine. The last dose of enalapril was administered in the evening of the day before surgery. General anesthesia was induced with propofol 120 mg, fentanyl 0.15 mg, and rocuronium 80 mg, followed by successful orotracheal intubation using a videolaryngoscope (McGrath® MAC video laryngoscope, Aircraft Medical, UK). Anesthesia was maintained with desflurane 5%, remifentanil 0.1 μg/kg/min, and an additional dose of fentanyl 0.05 mg and rocuronium 20 mg. After tracheal intubation, cefazolin 1 g was administered intravenously. Resection of her breast cancer was completed uneventfully in 111 min. Muscular relaxation was reversed by administering sugammadex 600 mg. The tracheal tube was removed after confirming her response, eye opening, and sufficient spontaneous breathing. Immediately after emergence from anesthesia, she was transferred to the intensive care unit (ICU). During anesthesia, the estimated blood loss was 40 mL, urine output was 230 mL, and 560 mL of crystalloids were infused. Around the completion of anesthesia, acetaminophen 1 g was administered for 15 min. Soon after admission to the ICU, famotidine 20 mg was administered.

**Fig. 1:**
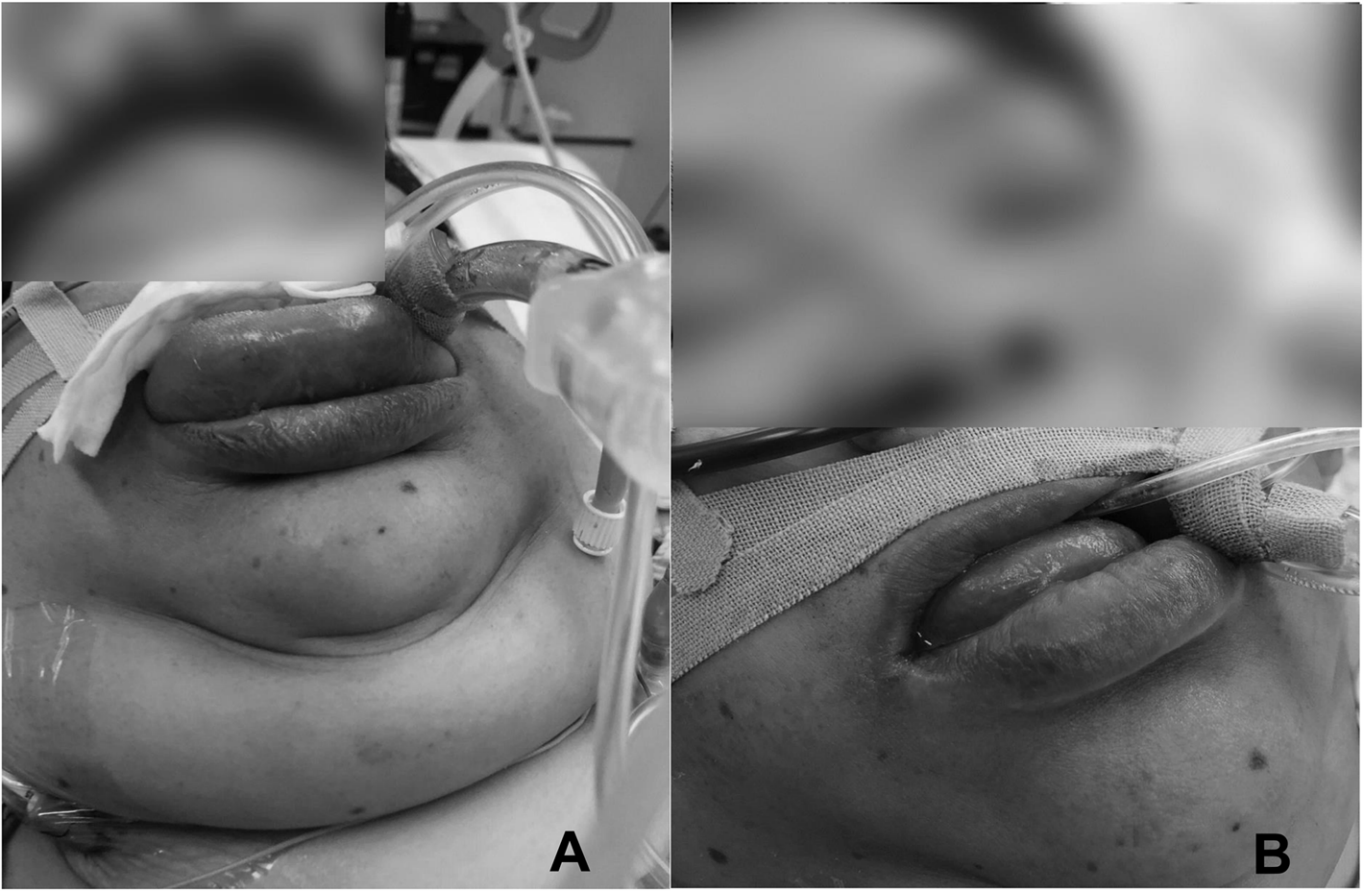
**a** The swollen tongue was protruding from the oral cavity after reintubation in the intensive care unit after surgery. **b** The massive tongue swelling was improved gradually on the next day.

Approximately 3 h after admission to the ICU, the patient complained of dyspnea and was rapidly desaturated over the next 15 min. She lost her consciousness and experienced airway obstruction and respiratory arrest. Immediately, manual mask ventilation was initiated; however, it was extremely difficult. Her tongue swelled heavily and protruded from the mouth. Tracheal intubation was attempted using a videolaryngoscope; however, the oral cavity was occupied with the swollen tongue. It was extremely difficult to determine the airway anatomical orientation. Her oxygen saturation decreased to 9%, and her heart rate dropped to 30 bpm. At this time, she regained her breath or started gasping respiration, which generated a faint bubble and revealed a possible airway. Fortunately, her airway was established using an ID 7.0-mm cuffed endotracheal tube without confirming the glottis or the vocal cord (Fig. 1a). At this juncture, we just considered ourselves to be extremely fortunate because emergent surgical airway establishment would have been frightfully difficult or impossible because of the patient’s physical appearance. Her oxygenation was improved up to 99–100%; no other allergic reactions such as urticaria, bronchospasm, and hemodynamic suppression were observed.

Because of localized edema, she was treated for angioedema. Dexamethasone 6.6 mg/day was tentatively administered for 3 days. During the next 2 days, her massive tongue swelling was improved gradually (Fig. 1b) and the tracheal tube was removed on the second postoperative day. The later course was uneventful. Laboratory blood test approximately 1 h after reintubation demonstrated normal levels of complement component 4, complement 1 inhibitor function, and complement 1q, suggesting that it was unlikely bradykinin-induced or hereditary angioedema [2, 3]. On the basis of these laboratory results, and because of the lack of family and past history, hereditary angioedema appeared to be negative. Topical infection was also negative in the view of clinical course. Therefore, we strongly suspected that it was due to drug-induced angioedema although other causes such as gene mutations, such as the *FXII* gene, should be excluded in advance [2, 3]. Incidentally, the total tryptase level in the same blood sample was also within the normal limit. Although the negative predictive value (NPV) for the clinical diagnosis of anaphylaxis was not so high (NPV = 0.4) [4], it was reasonable to assume that this event was not due to anaphylaxis considering the lack of other anaphylactic reactions. Lastly, her general practitioner was notified, and the adverse effect of the ACE inhibitor was documented in her medical record.

## Discussion

Angioedema is commonly mediated by histamine as a type I immunoglobulin E-mediated hypersensitivity to a variety of allergens (allergic angioedema) [2]. We initially ascribed angioedema in this case to famotidine or acetaminophen used in the ICU, because it occurred 3–4 h after surgery and for the first time in this patient. However, allergic angioedema is generally accompanied by other systemic allergic reactions such as urticaria [2], which was not observed in this patient, suggesting that it was unlikely an allergic reaction to agents used in the IUC or for anesthesia. Alternatively, we supposed bradykinin as a mediator of angioedema, based on previous reports showing that bradykinin-induced angioedema generally lacks urticaria and its onset is slower than allergic angioedema [2, 3].

ACE inhibitors are closely associated with bradykinin-related angioedema [2, 3]. It occurs from several hours to years after starting the use of ACE inhibitors. Of importance, ACE inhibitor-related angioedema occurs several months after its last dose. Our case had a long history of taking enalapril, an ACE inhibitor. Although it is difficult to determine the first episode of angioedema after starting the use of enalapril and the direct cause of it, previous reports also showed angioedema developed 15 min after extubation in a patient who started taking enalapril 10 days before surgery [4].

Although ACE inhibitors and angiotensin II receptor blockers (ARBs) contribute to improved renal outcomes [5], the blockade of the renin–angiotensin system does not have superiority over other antihypertensive medications to reduce cardiovascular and renal outcomes [6]. The possibility of inducing angioedema should always be considered particularly in the perioperative period and in morbidly obese patients. In this connection, we would like to discuss our difficult airway management in this case. We tried tracheal intubation using a videolaryngoscope under the situation that manual mask ventilation was extremely difficult. This “one best intubation” is advocated by the JSA difficult airway management guideline [7]. However, as this guideline recommends, the supraglottic airway devices might have allowed us to choose safer strategies [7]. To do so, it is very important that supraglottic airway devices are always ready to use even in the ICU.

We used only dexamethasone for treatment of angioedema. However, previous studies have shown that histamine H1 and H2 antagonists, corticosteroids, and adrenaline are unlikely effective for improving bradykinin-mediated angioedema [2, 3]. Dexamethasone might have also been ineffective in the present case, as shown by the prolonged time for extubation. Other treatments like fresh-frozen plasma, component 1 inhibitors, or icatibant, a bradykinin-2 receptor antagonist, might have facilitated the relief of symptoms [2, 3].

In conclusion, although ACE inhibitor-induced angioedema is rare, it is difficult to determine when and in whom it will occur. In addition, once it occurs, it can be potentially life threatening, especially for patients with possible difficult airway although there is still no evidence to support our message. In such patients, it has been suggested that marked obesity, resulting in airway narrowing, is a risk factor for upper airway obstruction secondary to ACE inhibitor-induced angioedema [8]. Considering the risk–benefit ratio, we must be more careful in administering ACE inhibitor therapy in morbidly obese patients.

## Data Availability

Not applicable

## Abbreviations

ACE: Angiotensin-converting enzyme
ICU: Intensive care unit
NPV: Negative predictive value
ARB: Angiotensin II receptor blocker

## References

[1] Langeron O, Birenbaum A, Le Saché F, Raux M (2014). Airway management in obese patient. Minerva Anestesiol..

[2] Bernstein JA, Cremonesi P, Hoffmann TK, Hollingsworth J (2017). Angioedema in the emergency department: a practical guide to differential diagnosis and management. Int J Emerg Med Int J Emerg Med..

[3] LoVerde D, Files DC, Krishnaswamy G (2017). Angioedema. Crit Care Med..

[4] Kharasch ED (1992). Angiotensin-converting enzyme inhibitor-induced angioedema associated with endotracheal intubation. Anesth Analg..

[5] Strippoli GF, Bonifati C, Craig M, Navaneethan SD, Craig JC. Angiotensin converting enzyme inhibitors and angiotensin II receptor antagonists for preventing the progression of diabetic kidney disease. Cochrane Database Syst Rev. 2006;2006:CD006257. 10.1002/14651858.CD006257 PMC6956646 17054288

[6] Bangalore S, Fakheri R, Toklu B, Messerli FH (2016). Diabetes mellitus as a compelling indication for use of renin angiotensin system blockers: systematic review and meta-analysis of randomized trials. BMJ..

[7] Japanese Society of Anesthesiologists (2014). JSA airway management guideline 2014: to improve the safety of induction of anesthesia. J Anesth..

[8] Jain M, Armstrong L, Hall J (1992). Predisposition to and late onset of upper airway obstruction following angiotensin-converting enzyme inhibitor therapy. Chest..

